# E2F1/CKS2/PTEN signaling axis regulates malignant phenotypes in pediatric retinoblastoma

**DOI:** 10.1038/s41419-022-05222-9

**Published:** 2022-09-12

**Authors:** Meng Chen, Zhaozhao Zhao, Lianqun Wu, Jiancheng Huang, Peng Yu, Jiang Qian, Ting Ni, Chen Zhao

**Affiliations:** 1 Eye Institute, Eye & ENT Hospital, Shanghai Medical College, Fudan University, Shanghai, China; 2 NHC Key Laboratory of Myopia (Fudan University), Key Laboratory of Myopia, Chinese Academy of Medical Sciences, and Shanghai Key Laboratory of Visual Impairment and Restoration (Fudan University), Shanghai, China; 3 State Key Laboratory of Genetic Engineering, Collaborative Innovation Center of Genetics and Development, Human Phenome Institute, School of Life Sciences, Fudan University, Shanghai, China

**Keywords:** Paediatric cancer, Paediatric cancer

## Abstract

Retinoblastoma (RB) is the most common pediatric intraocular malignancy and is a serious vision- and life-threatening disease. The biallelic mutation of the retinoblastoma gene *RB1* is the initial event in the malignant transformation of RB, but the exact molecular mechanism is still unclear. E2F transcription factors can be activated by *RB1* loss of function and lead to uncontrolled cell division. Among E2F family numbers, E2F1 has higher expression abundance than E2F2 and E2F3 in RB clinical samples. By integrating E2F1 ChIP-seq data, RNA-seq profiling from RB samples and RNA-seq profiling upon *E2F1* knockdown, together with pathway analysis, literature searching and experimental validation, we identified Cyclin-dependent kinases regulatory subunit 2 (*CKS2*) as a novel regulator in regulating tumor-associated phenotypes in RB. *CKS2* exhibited aberrantly higher expression in RB. Depletion of *CKS2* in Y79 retinoblastoma cell line led to reduced cell proliferation, delayed DNA replication and decreased clonogenic growth. Downregulation of *CKS2* also slowed tumor xenograft growth in nude mice. Importantly, reversed expression of *CKS2* rescued cancer-associated phenotypes. Mechanistically, transcription factor E2F1 enhanced *CKS2* expression through binding to its promoter and *CKS2* regulated the cancer-associated PI3K–AKT pathway. This study discovered E2F1/CKS2/PTEN signaling axis regulates malignant phenotypes in pediatric retinoblastoma, and CKS2 may serve as a potential therapeutic target for this disease.

## Introduction

Retinoblastoma (RB) is a rare pediatric intraocular malignancy arising from the biallelic mutation of the retinoblastoma gene (*RB1*) [1]. It is the only central nervous system cancer readily detected without any specialized equipment, can even be seen by the naked eye. Approximately 8000 children are diagnosed with RB each year worldwide [2, 3]. Globally, patient survival is associated with national income level (~30% in low-income countries [4, 5] and >95% in high-income countries [2, 6]). Lifelong follow-up is necessary since many patients with heritable retinoblastoma suffer secondary tumors [2, 7, 8].

*RB1* gene encodes a 928-amino-acid protein, pRB. pRB is well known as an essential cell cycle regulator that binds to E2F transcription factors (E2F1-3) and many other intermediaries to suppress cell-proliferation-associated genes [9–12]. Functional loss of *RB1* promotes cell from the G1 to S phase transition and leads to uncontrolled cell division [10, 13–15]. Biallelic inactivation of *RB1* is necessary but not sufficient for initiation of RB formation [2, 16]. *RB1* loss of function can be observed in most cancer types [17]. Although dysfunction of *RB1* is prevalent, the exact molecular mechanism for the malignant transformation of RB is still unclear and more genetic and epigenetic factors that are involved in this type of cancer remain to be discovered. Despite biallelic mutation of *RB1* existing in nearly all retinoblastomas, a subset of RB tumors (approximate ~1.4%) reveal no evidence of mutated *RB1* gene [18, 19] and possess high-level amplification of oncogene *MYCN*. Besides, this kind of amplified *MYCN* tumors have diverse morphology and patterns of gene expression different from RB1^−^^/−^ tumors, indicating a unique subtype [20].

Treatment of RB has undergone a series of changes, from initial systemic chemotherapy, to retention of the visual function, and removal of the eyeball. Nevertheless, comprehensive treatment based on systemic chemotherapy is still the most common clinical practice [21, 22]. Unlike some types of cancer, no targeted therapy has so far been applied to RB treatment. Understanding the pathogenesis of RB and its underlying molecular mechanism is still urgently needed to discover novel potential therapeutic targets for RB. Although several studies using microarray [23, 24] and RNA sequencing (RNA-seq) [25] techniques on a few RB samples have uncovered global changes in gene expression, the causal relationship between dysregulation and malignant phenotypes remains undetermined.

Here, we compared RNA-seq of a cohort of RB tumors and normal retinal samples. By screening a set of cancer-related genes directly regulated by transcription factor E2F1 and a series of experimental validations, we demonstrated that E2F1/CKS2/PTEN signaling axis regulates malignant phenotypes in RB and CKS2 may serve as a potential therapeutic target for retinoblastoma.

## Results

### Global transcriptional dysregulation in retinoblastoma

**Fig. 1:**
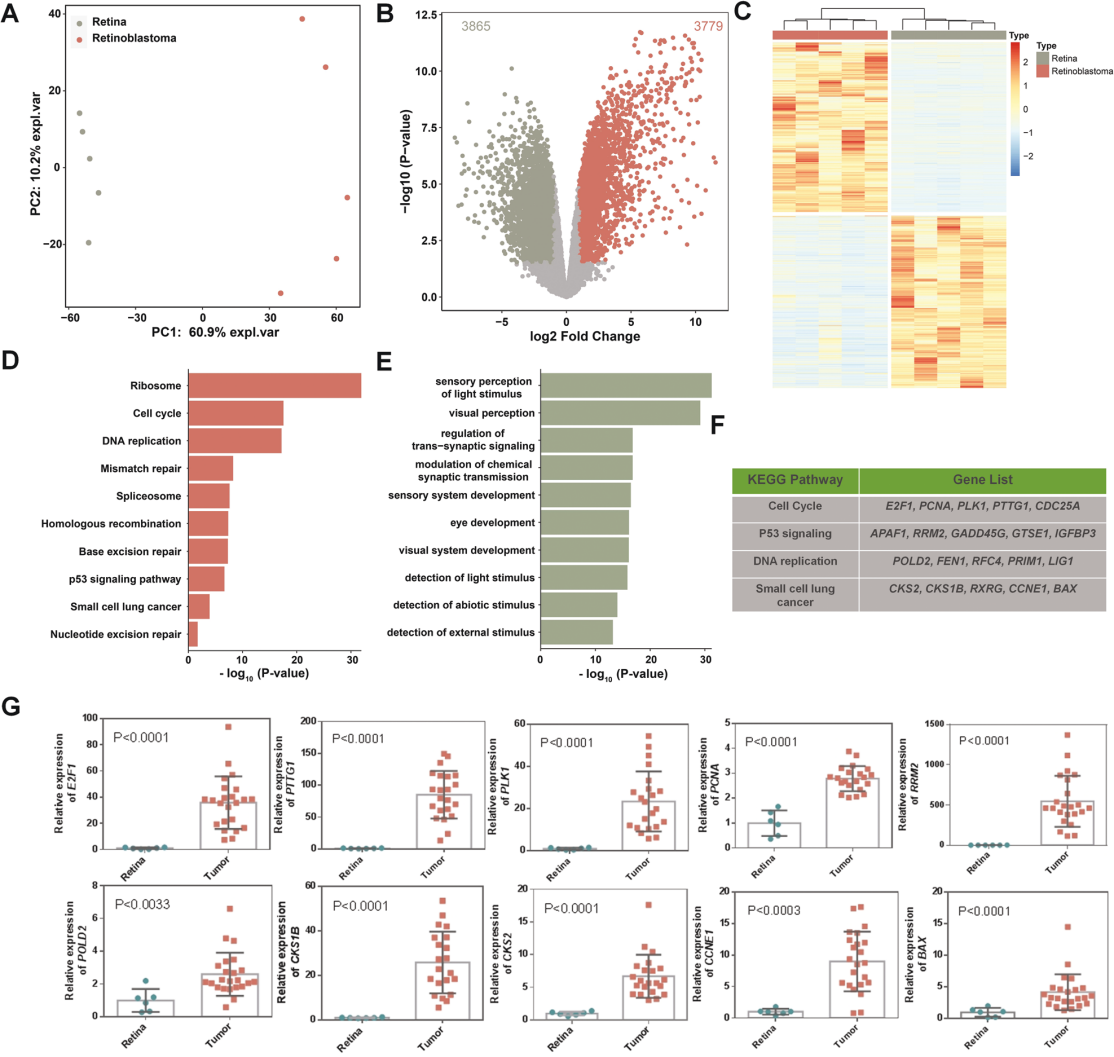
Global transcriptional dysregulation in retinoblastoma. **A** Scatter plot depiction of an unsupervised PCA of gene expression level from five RB and five retinal samples. **B** Volcano plot showing DEGs between RB and normal samples. The red and green dots represent genes with significantly increased or decreased expression in RB, respectively. **C** Heatmap of the expression profiles for the DEGs between RB and normal samples. Each column represents a sample and each line represents a DEG. **D** KEGG pathway analysis of the upregulated DEGs. Top annotation clusters are shown according to their enrichment scores [log^10^ (*p* value)]. Background genes were all stably expressed in these samples. *P* value was adjusted by Benjamini–Hochberg procedure. **E** GO analysis for downregulated DEGs. **F** Representative cancer-related pathways and their corresponding upregulated genes. **G** Ten cancer-associated genes were validated by qRT-PCR using clinical samples. Each dot represents a sample. ***P < 0.001; **P < 0.01; *P < 0.05, by two-tailed *t*-test.

To determine the differences in mRNA expression level between RB and retina, the transcriptomes of five RB and five normal retina samples were analyzed using RNA-seq. To confirm the reliability of pathogenic and normal tissues, principal component analysis (PCA) was applied to these ten samples. The result showed a clear distinction between the tumor and non-tumor samples (Fig. 1A), suggesting that the transcriptome contained important information to distinguish these two conditions. The separation of tumor and non-tumor samples remained true when we combined our expression profiles with Rajasekaran’s dataset [25], the first in-depth RNA-seq resource of retinoblastoma (Supplemental Fig. S1A, B). Next, we explored differentially expressed genes (DEGs) between RB and normal controls. 3779 upregulated and 3865 downregulated DEGs were identified in tumor samples (Fig. 1B, C). Gene Ontology (GO) and Kyoto Encyclopedia of Genes and Genomes (KEGG) pathway analyses revealed that upregulated genes in tumor samples were enriched in pathways including cell cycle, DNA replication, and other cancer-associated pathways (Fig. 1D), and downregulated genes were enriched in pathways related to light stimulus and eye development, which are retina specific (Fig. 1E). Some cancer-related pathways and corresponding upregulated genes were listed in Fig. 1F. Ten cancer-associated genes were randomly chosen and their differential expression levels were validated by reverse transcription followed by quantitative polymerase chain reaction (qRT-PCR; Fig. 1G) using clinical samples, in line with the results in RNA-seq data (Supplemental Fig. S2). These results indicate that RB undergoes dramatic transcriptomic changes, some of which are in cancer-associated genes.

### Screening of cancer-related genes directly regulated by transcription factor E2F1

**Fig. 2:**
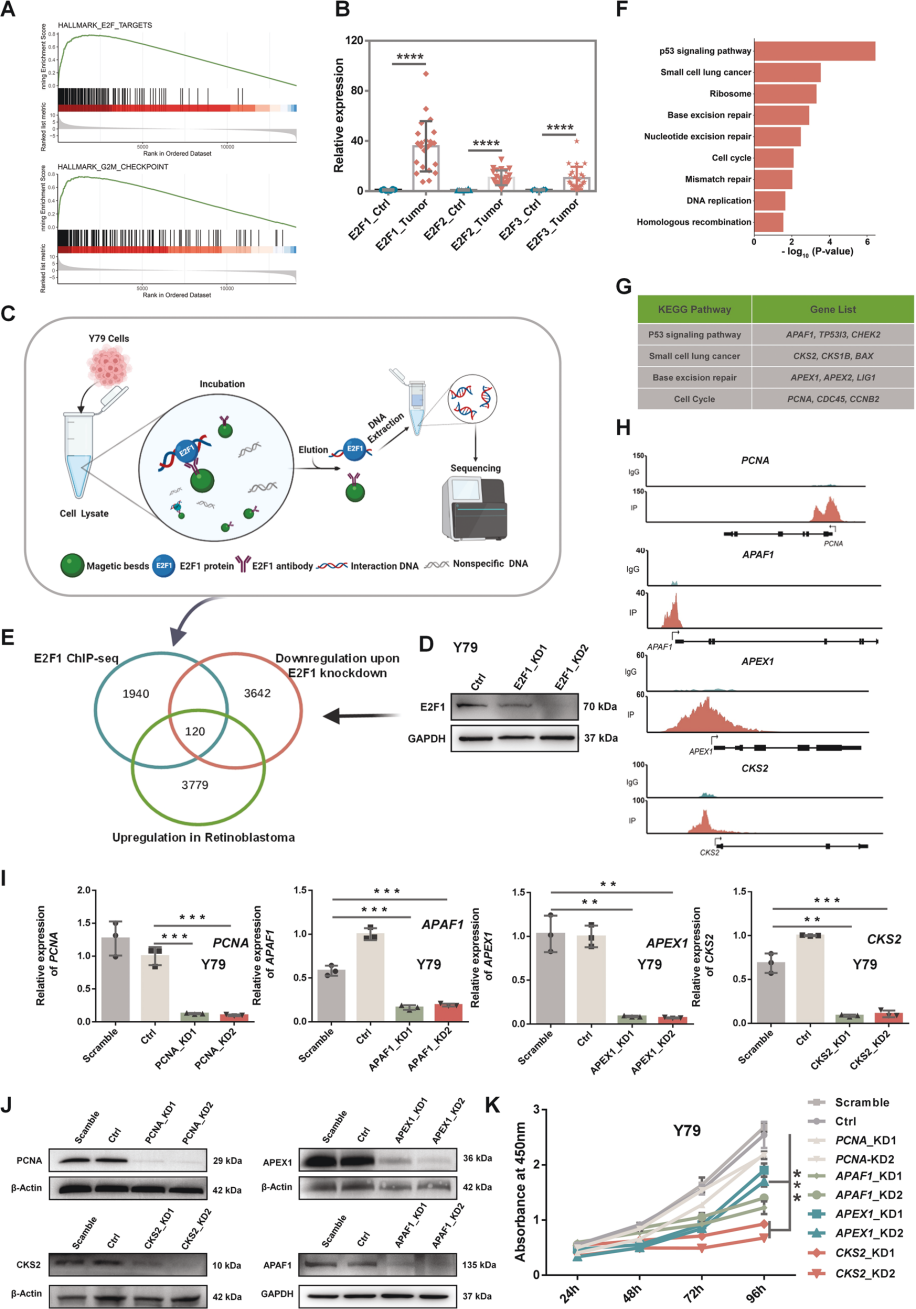
Screening cancer-related genes directly regulated by transcription factor E2F1. **A** Representative GSEA plots showing that upregulated DEGs were enriched in E2F targets (top) and the G2M checkpoint (bottom) in the RB versus retinal samples. **B** E2F1-3 were validated by qRT-PCR using clinical samples, in line with blue dot (normal tissues) and red dot (RB samples). **C** Schematic diagram of E2F1 ChIP-seq in Y79 cells. **D** Knockdown efficiency of E2F1 was evaluated by western blotting. **E** The diagram of overlapping gene numbers among E2F1 ChIP-seq profiling, RNA-seq profiling of E2F1-KD (down-regulated genes) and RNA-seq profiling of RB (upregulated genes), created with BioRender website. **F**, **G** The pathway analysis of cancer-related genes directly regulated by E2F1 **(F)** and their corresponding upregulated genes **(G)**. **H** ChIP-seq tracks of four representative genes. **I**, **J** Four representative candidate genes showing downregulation by Lentivirus knockdown in Y79 cells were evaluated by qRT-PCR **(I)** and western blotting **(J)**. GAPDH or beta Actin served as an internal control. **K** Proliferation rate of Y79 cells without (Scramble and Ctrl) and with gene downregulation (*PCNA*, *APAF1*, *APEX1*, *CKS2*) were analyzed by a Cell Counting Kit-8 (CCK-8) assay. ***P < 0.001; **P < 0.01; *P < 0.05, by two-tailed *t*-test.

It is widely known that E2F transcription factors (E2F1-3) are activated by *RB1* loss of function and augment expression of cell-proliferation-associated genes leading to uncontrolled cell division [9–15]. Gene set enrichment analysis (GSEA) using our RNA-seq data showed that the upregulated genes were related to DNA repair and cell cycle progression (Supplemental Fig. S3A–D). The upregulated genes in RB were enriched in E2F targets and the G2M checkpoint gene set (Fig. 2A), consistent with previous knowledge [9–15]. We validated expression of E2F family members (E2F1-3) by qRT-PCR using clinical samples. Result showed that *E2F1* had higher expression level than *E2F2* and *E2F3* (Fig. 2B), implying that *E2F1* might play a more important role than the other two family members in malignant transformation. Following this clue, screening cancer-related genes directly regulated by transcription factor E2F1 was the crucial step to explore the underlying mechanism of malignant phenotypes in RB. In order to achieve this purpose, we performed E2F1 chromatin immunoprecipitation sequencing (ChIP-seq) in Y79 retinoblastoma cell line (E2F1 ChIP-seq schematic diagram shown at Fig. 2C). RNA-seq libraries before and after knocking down (KD) E2F1 in the same cell line were also constructed and sequenced. Knockdown efficiency of E2F1 was evaluated by Western blotting (Fig. 2D). By overlapping three groups of genes, including those having E2F1 ChIP-seq peaks in Y79 cells (directly bound by E2F1 at the promotor area), those showing downregulation upon E2F1 knockdown in Y79 cells (potentially regulated by E2F1 at the level of transcription) and those exhibiting upregulation in RB samples compared with normal controls (upregulate in RB clinical samples), we found that 120 genes were potentially bound and directly regulated by E2F1 in RB cells (Fig. 2E). Pathway enrichment analysis revealed that those genes were enriched in cancer-relevant pathways, including p53 signaling pathway, small cell lung cancer, cell cycle and base excision repair (Fig. 2F). Some corresponding genes were listed in Fig. 2G. Four genes that were relatively highly expressed from each term were selected for initial functional screening and ChIP-seq peaks of four representative genes showed strong binging of E2F1 near the promoter regions (Fig. 2H). Lentivirus knockdown vectors for these four genes were constructed and transfected to Y79 cells. Knockdown of corresponding genes was confirmed by qRT-PCR (Fig. 2I; Supplemental Table S1) and Western blotting (Fig. 2J). To determine which gene had the greatest effect on progression of malignant transformation of RB, we measured proliferation rate changes in Y79 cells. As *CKS2* exhibited the greatest impact on slowing the proliferation rate than the other genes when knocking down (Fig. 2K), we focused on *CKS2* to explore its function and mechanism in RB.

### Transcription factor E2F1 enhances CKS2 expression through binding to its promotor

**Fig. 3:**
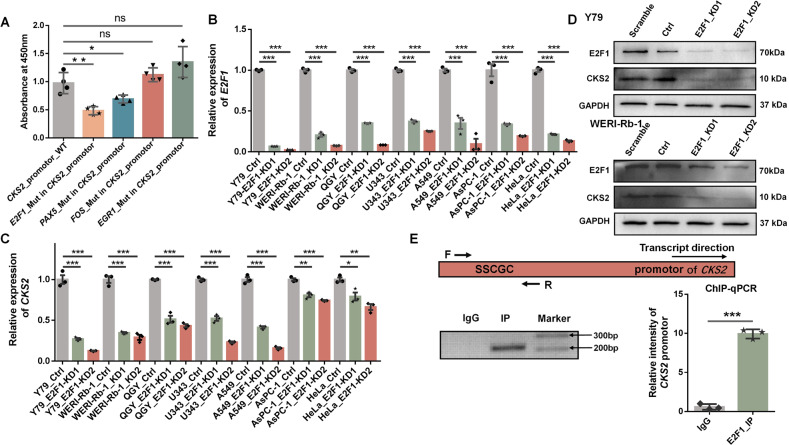
E2F1 binds to *CKS2* promotor and regulates its expression. **A** Luciferase activity of *CKS2*’s wild-type (WT) and mutated (Mut) promotors in Y79 cells. The mutation was achieved by replacing a palindromic sequence to a specific TF potential binding site. **B** Knockdown of *E2F1* in Y79, WERI-Rb-1, QGY, U343, A549, AsPC-1 and HeLa human cells evaluated by qRT-PCR. *GAPDH* served as the internal control. **C** Validation of *CKS2* expression after *E2F1* knockdown in Y79, WERI-Rb-1, QGY, U343, A549, AsPC-1 and HeLa human cells evaluated by qRT-PCR. *GAPDH* served as the internal control. **D** Western blotting showed that the protein expression of *CKS2* was significantly reduced in *E2F1*-KD Y79 (up panel) and WERI-Rb-1 (down panel) cells. GAPDH served as the internal control. **E** E2F1 binding to the promotor region of *CKS2* was evaluated by ChIP-PCR and ChIP-qPCR in Y79 cells. The black arrow represents the transcription direction. The ChIP-PCR and ChIP-qPCR primer pairs (F and R, forward and reverse, respectively) were designed spanning the “SSCGC” motif (S = G or C) within the ChIP-seq peak region. The same amount of DNA was applied for ChIP-PCR (left panel) and ChIP-qPCR (right panel). ***P < 0.001; **P < 0.01; *P < 0.05, by two-tailed *t*-test.

To explore which transcription factor (TF) potentially bound to the promoter region of *CKS2*, different databases were used including PROMO [26], USCS Genome Browser [27], ISMARA [28] and JASPAR [29]. We speculated that the potentially functional TFs should be differentially expressed between RB and normal controls in our RB clinical RNA-seq data (Supplemental Fig. S4). The potential binding sites of *CKS2* promotor region with different TFs were predicted by ChIPBase v2.0 (http://rna.sysu.edu.cn/chipbase) [30]. Four transcription factors, including E2F1, FOS, PAX5 and EGR1, were selected as candidates. To evaluate whether these candidate TFs could bind to the promoter region of *CKS2*, a dual-luciferase vector psiCHECK2 with *CKS2*’s wild-type (WT) or mutated promotors (Mut), was constructed, and then was transfected into Y79 cells for comparison. TFs predicted binding sites in *CKS2*’s promotor were replaced by a palindromic sequence (Shown at Supplemental Fig. S5). Vector with mutation at E2F1 binding sites (*E2F1*_Mut in *CKS2*_promotor) showed significantly reduced luciferase activity than WT and other mutant groups (*FOS*_Mut in *CKS2*_promotor, *PAX5*_Mut in *CKS2*_promotor and *EGR1*_Mut in *CKS2*_promotor) (Fig. 3A), indicating that TF E2F1 might directly bind to *CKS2*’s promotor region to regulate its target gene’s expression. We also observed the similar result when vectors were transfected into 293T cells (Supplemental Fig. S6).To further explore the relationship between *E2F1* and *CKS2*, based on online tool GENPIA [31], which integrates RNA-seq data from the Cancer Genome Atlas (TCGA), we observed that both *E2F1* and *CKS2* were highly expressed compared to the normal tissues in 24 and 23 cancer types, respectively (Supplemental Figs. S7 and S8). *E2F1* and *CKS2* were both highly expressed in 21 cancer types (Supplemental Figs. S7 and S8) and such positive correlation was also observed in our RB clinical data (Fig. 1G). To confirm that E2F1 did play a causal role in promoting transcription of *CKS2*, we knocked down E2F1 with two short hairpin RNAs (shRNAs) in human retinoblastoma cells (Y79 and WERI-Rb-1) and found that expression of *CKS2* was significantly decreased in both RNA and protein levels (Fig. 3B–D). We also knocked down E2F1 in other cancer cell lines (human hepatocellular carcinoma, QGY-7701; human glioma, U343; human non-small-cell lung carcinoma, A549; human pancreatic carcinoma, AsPC-1; human cervical carcinoma, HeLa) in which *E2F1* and *CKS2* were both highly expressed based on TCGA database (Supplemental Figs. S7 and S8) and exhibited positive co-expression between *E2F1* and *CKS2* (Fig. 3B, C). The lines of evidence showed that expression of *CKS2* reduced correspondingly upon knockdown of E2F1 (Fig. 3A–D). Further, we used ChIP Base v2.0 to uncover that the potential binding site of E2F1 located within one kilobase (kb) upstream of *CKS2* transcription start site and ChIP-seq peaks were also displayed in this region containing a canonical E2F binding site (SSCGC with S = C or G) [32] using our ChIP-seq data (Y79 cells) and ChIP Base v2.0 data (MCF7 cells) [30] (Fig. 2H; Fig. 3E; Supplemental Fig. S9), supporting the potential binding of E2F1 to the promoter of *CKS2*. To confirm the direct binding, we performed ChIP coupled with PCR (ChIP-PCR) and quantitative PCR (ChIP-qPCR) assay in Y79 cell line. The ChIP-PCR and ChIP-qPCR primer pair was designed spanning the “SSCGC” motif (S = G or C) within the ChIP-seq peak region (Fig. 3E; Supplemental Table S1). The result manifested an enrichment of E2F1 binding signals to this region when compared with a non-specific IgG control (Fig. 3E). Together, these lines of evidence indicate that E2F1 directly binds to the promotor of *CKS2* to regulate its expression.

### CKS2 exhibits aberrant higher expression in retinoblastoma and promotes cell proliferation and tumor formation

**Fig. 4:**
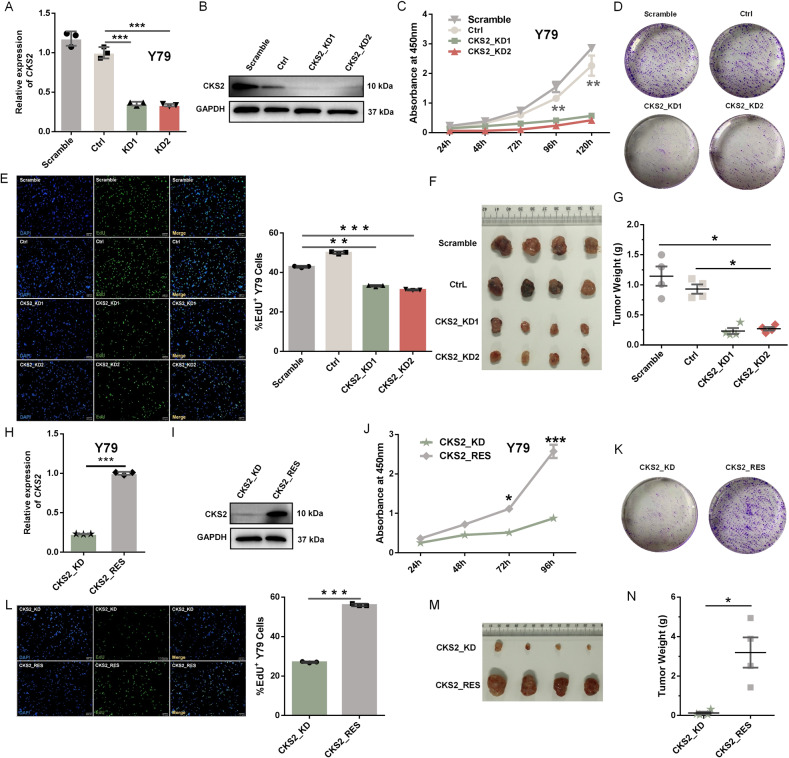
Expression of *CKS2* contributes to cancer-associated phenotypes. **A**, **B** Lentivirus knockdown of *CKS2* in Y79 cells evaluated by qRT-PCR **(A)** and western blotting **(B)**. *GAPDH* served as the internal control. **C** Proliferation rate evaluation of Y79 without (Scramble and Ctrl) and with *CKS2* knockdown (CKS2_KD1 and CKS2_KD2) by CCK-8 assay. **D** Y79 cells were seeded in six-well plates at 1500 cells per well for colony formation assay. **E** Left panel: Representative images of EdU staining assay of Y79 cells without (Scramble and Ctrl) and with CKS2 knockdown (CKS2_KD1 and CKS2_KD2). Right panel: Quantification of percent EdU^+^ cells, shown as mean ± SEM, **p < 0.01, ***p < 0.001, (*n* ≥ 3). **F, G** Tumor xenografts using Y79 cells in *CKS2* knockdown groups (*CKS2*_KD1 and CKS2_KD2) were smaller than without CKS2-KD groups (Scramble and Ctrl). Representative photographic images (**F**) and tumor weights (**G**) were shown (each group *n* = 4). **H**, **I** Lentivirus overexpression of *CKS2* in *CKS2*-KD cells (*CKS2*_Res) restored expression of *CKS2* in both RNA **(H)** and protein **(I)** levels. *GAPDH* served as the internal control. **J** Proliferation rate evaluation of Y79 cells between CKS2_KD group and CKS2_RES (Rescue) group by CCK-8 assay. **K, L** Colony formation assay **(K)** and EdU staining assay **(L**; Representative images at left panel and quantification of percent EdU^+^ cells at right panel^,^ shown as mean ± SEM, **p < 0.01, ***p < 0.001, *n* ≥ 3) of Y79 cells between CKS2_KD group and rescue group. **M, N** Tumor formation evaluation of Y79 cells between CKS2_KD group and CKS2_RES group. Representative photographic images (**M**) and tumor weights **(N)** were shown (each group *n* = 4). ***P < 0.001; **P < 0.01; *P < 0.05, by two-tailed *t*-test.

After demonstrating that *CKS2* had aberrantly higher expression in RB samples compared to normal controls (Fig. 1G), we asked whether *CKS2* contributed to cancer-associated phenotypes. To test this, we knocked down *CKS2* with two shRNAs in Y79 cells (Supplemental Table S1). Knockdown efficiency of *CKS2* in Y79 cells was evaluated by qRT-PCR and Western blotting (Fig. 4A, B). CCK-8 assay suggested that downregulation of *CKS2* led to delayed cell proliferation (Fig. 4C). Cell colony formation assay demonstrated that *CKS2* depletion repressed clonogenic growth of Y79 cells (Fig. 4D). EdU staining indicated that KD of *CKS2* resulted in decreased DNA replication rate (Fig. 4E). Depletion of *CKS2* delayed tumor xenograft growth of Y79 cells in nude mice (Fig. 4F). The mean tumor weight of the CKS2_KD group was significantly reduced (Fig. 4G). Moreover, recovery of *CKS2* by lentivirus-mediated overexpression of *CKS2* in *CKS2*-KD cells (CKS2_Rescue) restored expression of *CKS2* in both RNA and protein levels (Fig. 4H, I) and rescued cancer-associated phenotypes including cell proliferation, colony formation, EdU staining and tumor xenograft growth (Fig. 4J–N). We also knocked down *CKS2* with two shRNAs in human retinoblastoma WERI-Rb-1 cells (Supplemental Fig. S10A) and found that downregulation of *CKS2* in this cell type led to similar phenotypes as well including cell proliferation, colony formation and EdU staining (Supplemental Fig. S10B–D). These results indicate that *CKS2* is a novel contributor to cancer-related phenotypes in RB.

### CKS2 regulates cancer-associated PI3K–AKT pathway

**Fig. 5:**
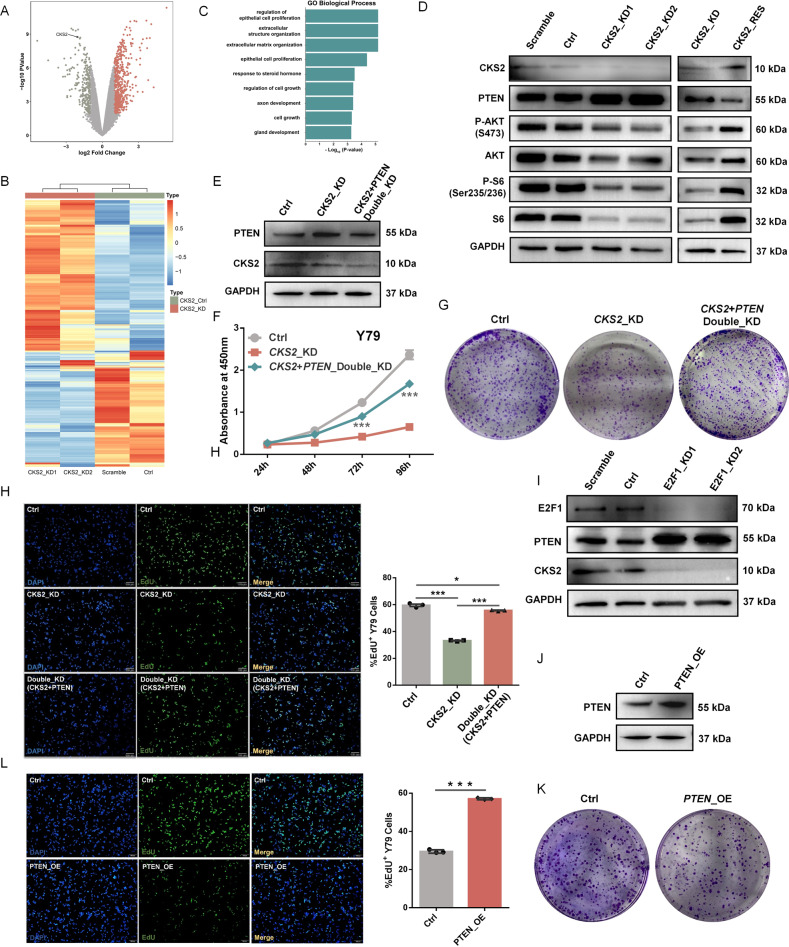
*CKS2* regulates cancer-associated PI3K–AKT signaling pathway. **A** Volcano plot of gene-level expression changes after *CKS2* knockdown. The orange and green dots represent genes with an at least two-fold increase or decrease in expression level in *CKS2*-KD cells compared with control cells. **B** Heatmap of log2-transformed expression profiles for DEGs compared *CKS2*-KD cells with control cells. Orange and blue denote increased and decreased expression, respectively. **C** GO enrichment analysis for all DEGs showed in **(C)**. **D** Western blotting validations of PI3K–AKT–mTOR signaling pathway-associated proteins in *CKS2*-KD and *CKS2*-Rescue cells compared with corresponding control Y79 cells. **E** Western blotting was used to validate the knockdown of *PTEN* in *CKS2* down-regulated Y79 cells. **F**–**H** Knockdown of *PTEN* in *CKS2* down-regulated Y79 cells reversed cell proliferation (**F**), colony formation **(G)** and EdU staining assay **(H**, Representative images at left panel and quantification of percent EdU^+^ cells at right panel, shown as mean ± SEM, **p < 0.01, ***p < 0.001, *n* ≥ 3). **I** Western blotting was used to validate expression of *PTEN* in *E2F1* knockdown Y79 cells. **J** Lentivirus overexpression of *PTEN* (*PTEN*-OE) in Y79 cells was validated by Western blotting. GAPDH served as the internal control. **K, L** Colony formation assay **(K)** and EdU staining assay (**L**; Representative images at left panel and quantification of percent EdU^+^ cells at right panel, shown as mean ± SEM, **p < 0.01, ***p < 0.001, *n* ≥ 3) of Y79 cells between Ctrl group and *PTEN*-OE group. ***P < 0.001; **P < 0.01; *P < 0.05, by two-tailed *t*-test.

To explore the downstream signaling pathway of *CKS2* in explaining the malignant phenotypes of RB, RNA-seq analyses were performed in Y79 cells under scramble, control, CKS2-KD1 and CKS2-KD2 conditions (for the vectors construction, see details in the “Method” section). Gene expression analysis found 566 upregulated and 257 down-regulated genes in *CKS2*-KD cells (Fig. 5A, B; fold change > 2, false discovery rate (FDR) < 0.05). GO analysis identified enrichment of some proliferation-related biological processes among *CKS2*-dependent genes including upregulated and downregulated genes (Fig. 5C), in line with the reduced proliferation rate (Fig. 4C–G). *PTEN*, a well-known tumor suppressor gene and the most crucial negative regulator of the PI3K–AKT signaling pathway [33–36], showed an increased protein level in *CKS2*-KD cells (Fig. 5D) in line with the result of *CKS2*-KD RNA-seq data (Supplemental Fig. S11). Rescue *CKS2* by lentivirus-mediated overexpression of *CKS2* in *CKS2*-KD cells reversed *PTEN* protein level (Fig. 5D), indicating that *CKS2* acts upstream of *PTEN*. We also quantified two key proteins (AKT and S6) and their phosphorylation states in the PI3K–AKT–mTOR signaling pathway and found that phosphorylation of AKT (p-AKT) and S6 (p-S6) both decreased upon *CKS2* knockdown (Fig. 5D). Recovery of *CKS2* by lentivirus-mediated overexpression of *CKS2* in *CKS2*-KD cells significantly reversed the levels of p-AKT and p-S6 (Fig. 5D). Knockdown of PTEN in *CKS2*-KD cells did not impair *CKS2* protein level, but partially rescued cancer-associated phenotypes including cell proliferation, colony formation and EdU staining (Fig. 5E–H), suggesting that *PTEN* act downstream of *CKS2*. To validate that *PTEN* was indeed downstream of RB-E2F1, we observed that E2F1 knockdown had the same effect of *CKS2* depletion on *PTEN* protein levels in Y79 cells (Fig. 5D, I). Moreover, overexpression of PTEN in Y79 cells suppressed the cell proliferation independently including colony formation and EdU staining (Fig. 5J–L). These data support that *CKS2* can affect the activity of the PI3K–AKT pathway and may explain why *CKS2* regulates the retinoblastoma-associated phenotypes.

## Discussion

Retinoblastoma is a rare type of intraocular malignancy that usually develops in early childhood, and is a serious vision- and life-threatening disease. Previous few microarray [23, 24] and RNA-seq [25] studies have uncovered global gene expression changes between RB and control samples. However, lack of RB samples without chemotherapy and normal retinal samples from young donors as age-matched controls are two possible restrictions to global gene-expression research for RB. Additionally, these transcriptomic analyses have only discovered an association but not causality between gene expression and RB. The previous RNA-seq study used six RB samples and two control samples (age at 12 and 22) [25]. In our study, we sequenced five RB samples and five control samples. As age-matched normal samples are difficult to obtain since most RB patients are age under five years, the normal retinal samples used in this study were from people aged 17–40 years. If age is the major contributor to gene expression, we would expect the first principal component (PC) regarding expression profiling among all these samples (both RB and normal retina) would show an age-dependent manner. However, when carrying out principal components analysis (PCA), we found that the normal retinal and RB samples could be separated very well (Fig. 1A), suggesting that tumorigenesis was the major contributor of the transcriptome difference. Combing previously published RNA-seq data into our datasets showed similar results (Supplemental Fig. S1), supporting the speculation.

A recent long non-coding RNA (lncRNA) study uncovered that a novel transcript, RBAT1, accelerates tumorigenesis in RB through recruiting HNPNPL and cis-activating E2F3 [37]. And beyond that, to the best of our knowledge, no disease-causing genes have been discovered based on transcriptomic analysis of RB samples. To demonstrate that RNA-seq data could be valuable for causality studies of RB, we integrated transcriptomic analysis, gene functional enrichment, literature searching, experimental validation, and gene perturbation strategy to screen a novel cancer-causing gene *CKS2* in RB. We found that elevated *CKS2* expression was prevalent in RB samples. Upregulation of *CKS2* promoted cell proliferation and tumor formation while depletion of *CKS2* led to reduced cell proliferation, delayed DNA replication and decreased clonogenic growth. In addition to the causality demonstration, we inferred that the cancer-associated PI3K–AKT–mTOR pathway could potentially explain *CKS2*-mediated cancer phenotypes. Inhibitors of PI3K–AKT–mTOR, such as rapamycin and LY294002 [38–41], could be evaluated for their potential utility in mouse experiments and clinical trials for treatment of RB patients, and deserved further investigation.

**Fig. 6:**
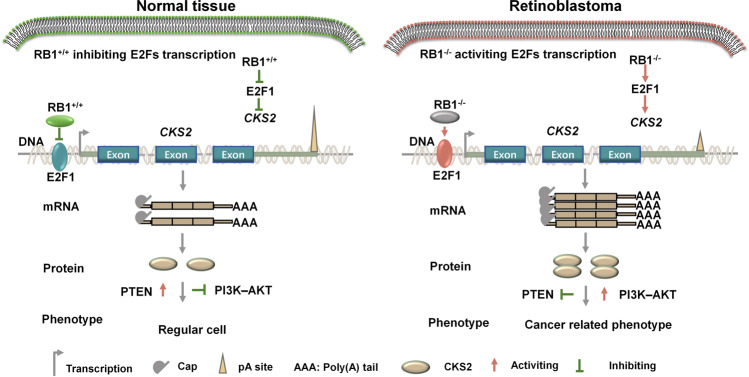
Working model for E2F1/CKS2/PTEN signaling axis in regulating malignant phenotypes of RB. *CKS2* exhibits aberrant higher expression in retinoblastoma compared with normal controls. Upregulated transcription factor E2F1 bound to upstream of *CKS2* transcription start site and promoted CKS2 protein production, leading to cancer-associated phenotypes, possibly by regulating PI3K–AKT signaling pathway.

Mechanistically, transcription factor E2F1 could be one of the upstream regulators of *CKS2*. Since most RB patients have *RB1* gene mutation, to establish the bridge between *CKS2* upregulation and *RB1* gene inactivation is important. E2F1 could be this linkage, because a well-known knowledge is that *RB1* inactivation elevates E2Fs expression and leads to uncontrolled cell proliferation [9–12, 14, 15]. Besides, based on GENPIA, *E2F1* and *CKS2* had significantly positive co-expression in 21 of 33 types of cancer (Supplemental Figs. S6 and S7), which might be a possible reason why *CKS2* always frequently elevated in many cancers. Working model for *CKS2* as a novel regulator in regulating malignant phenotypes of RB was shown at Fig. 6.

In conclusion, our study uncovered the crucial role of E2F1/CKS2/PTEN signaling axis in regulating malignant phenotypes of RB and *CKS2* might become a novel therapeutic target for RB in the future.

## Materials and methods

### Clinical sample collection

A total of ten clinical samples were obtained from Eye & ENT Hospital of Fudan University, Shanghai, China. Five normal retinal samples were acquired from donors aged 17–40 years. Five RB samples were collected from patients aged 1–4 years who had not received radiotherapy, chemotherapy or other related therapies before surgery. The diagnosis was validated according to clinical manifestation and pathological examination. The whole process was approved by Eye & ENT Hospital of Fudan University.

### RNA-seq data analysis

The raw paired-end reads obtained from RNA-seq experiments were filtering out low-quality reads, and then aligned to human reference genome sequence (UCSC hg19 assembly) using STAR [42] with default settings. To choose genes with accurate expression value, we consider genes whose FPKM (fragments per kilobase of exon model per million reads mapped) > 1 in at least one sample for subsequent analysis. Differentially expressed gene analysis was performed using edgeR [43] and a statistical cutoff of FDR < 0.05 and fold change > 2 was applied to obtain regulated genes. GO analysis, KEGG analysis and gene set enrichment analysis (GSEA) were performed by clusterProfiler [44]. Hallmark gene signature sets maintained by the Molecular Signatures Database (MSigDB) [45] were used.

### Cell culture, vector construction and transfection

Human 293T, HeLa, QGY, U343, A549, AsPC-1, Y79 and WERI-Rb-1 cells were cultured in Dulbecco’s modified Eagle’s medium (DMEM) or RPMI 1640 medium with 10% fetal bovine serum (FBS) at 37 °C in a 5% CO_2_ incubator. For *PCNA*, *APAF1*, *APEX1*, *CKS2* and *E2F1-KD* vector construction, two pair of annealed shRNA oligonucleotides were cloned into pLKO.1 plasmid for each gene with *Eco*RI and *Age*I restriction enzymes. A pair of shRNAs (Supplemental Table S1) Non-targeting a known gene was used as scramble and pLKO.1 empty vector that transfected into Y79 cells served as control. To obtain *CKS2* and *PTEN* overexpression vector, the full coding sequences without stop codon sequence was inserted into PCDH_EF1_MCS_T2A_Puro vector (PCDH). PCDH empty vector transfected into target cells served as overexpression experiment control. For lentivirus transduction, 293T cells were seeded in six-well plates with approximately 70% confluence and transfected with vectors using Lipofectamine 3000 (Thermo Fisher Scientific). After 24 h (h) culture, supernatant of the virus was collected to infect Y79 and other target cells (WERI-Rb-1, QGY, U343, A549, AsPC-1 and HeLa). To achieve stably transformed cells, 1640 or DMEM medium with 3 μg/ml puromycin was used for cell culture.

### qRT-PCR and western blotting

Total RNA and protein of each sample were extracted using TRIzol reagent (Ambion), and reversely transcribed into cDNA using random primers. Expression of the tested genes was quantified by qRT-PCR using 2*ChamQ Universal SYBR qPCR Master Mix and normalized to *GAPDH* (Roche Light Cycler). All primer sequence information is listed in Supplemental Table S1. The protein concentration was evaluated by BCA protein assay kit (Vayme Biotechnology). Primary antibodies involved in CKS2 (Abcam, cat.No: ab155078, 1:1000), APEX1 (Proteintech, cat.No: 10203-1-AP, 1:1000), APAF1 (Cell Signaling Technology, cat.No: #8969, 1:1000), PCNA (Arigo, cat.No: ARG62605, 1:5000), GAPDH ((Proteintech, cat.No: HRP-60004, 1:5000), β-Actin (Proteintech, cat.No: HRP-60008, 1:5000), E2F1 (Abcam, cat.No: ab179445, 1:1000), AKT (Cell Signaling Technology, cat.No: 4691, 1:1000), p-AKT (Cell Signaling Technology, cat.No: 4060, 1:1000), S6 (Cell Signaling Technology, cat.No: 2217, 1:1000), p-S6 (Cell Signaling Technology, cat.No: 2211, 1:1000), and PTEN (Proteintech, cat.No: 22034-1-AP, 1:1000) were used for Western blotting. Full length western blot scans for the cropped images presented in supplemental material file.

### Colony formation assay

To assess the colony formation ability of the RB cell line (Y79 and WERI-Rb-1) with or without gene perturbation, six-well plates were treated with 0.1 mg/ml poly-L-lysine for 2 h and target cells were seeded at 1500 cells per well with RPMI 1640 medium with 10% FBS. After ten days of cell culture, cells were fixed in 4% paraformaldehyde for 30 min (min) and stained with crystal violet staining solution for 1 h. Different wells with or without *CKS2* perturbed cell clones were taken pictures after staining.

### Cell proliferating rate assay/CCK-8 assay

CCK-8 is a simple, rapid, sensitive colorimetric assay for the evaluation of the number of viable cells. Y79 cells with or without *CKS2* gene perturbation were cultured and assayed for cell proliferation using the CCK-8 kit according to the protocol provided by vendor Dojindo.

### EdU staining assay

Validation of DNA replication was performed according to the protocol of KeyFluor488 Click-iT EdU Kit (KeyGEN Biotechnology). Briefly, 24-well plates were treated with 0.1 mg/ml poly-L-lysine for 2 h, and Y79 cells with or without *CKS2* gene perturbation were seeded to approximate 60% confluence. After cells adhered, 4% formaldehyde was used to treat the cells for 30 min. After three washes with 3% bovine serum albumin in phosphate-buffered saline, cells were incubated with staining solution for 2 h in the dark. After EdU staining, cell nuclei were stained with 5 μg/ml 4′,6-diamidino-2-phenylindole solution at room temperature for 30 min, and then analyzed by inverted fluorescence microscopy (Olympus IX73).

### Tumor xenograft model

Animal experiments were approved by the Institutional Research Ethics Committee of Eye & ENT Hospital of Fudan University. Y79 cells (1 × 10^7^) with or without *CKS2* gene perturbation were subcutaneously injected into 5-week-old nude mice (each group *n* = 4). Tumor volume was monitored by calipers every two days and calculated according to the formula: tumor volume = (length × width^2^)/2. After 5 weeks, all nude mice were sacrificed, and tumor xenografts were dissected and weighed.

### Luciferase assays

To evaluate whether target of TFs could bind to the promoter region of *CKS2*, a dual luciferase vector psiCHECK2 (Promega, cat. no. C8021) with *CKS2*’s wild-type (WT) or mutated promotors (Mut) was constructed. The promotor (WT or Mut) of *CKS2* was cloned into the downstream of the *Renilla* luciferase translational stop codon using the XhoI and PmeI restriction enzyme sites, and the firefly reporter cassette served as an intra-plasmid transfection normalization reporter. After 24-well plates treated with 0.1 mg/ml poly-L-lysine for 2 h, Y79 cells were seeded into a 24-well plate at approximate 60% confluence. After overnight, the cells were transfected with psiCHECK2 vectors (*CKS2*’s WT or mutated promotors) using Lipofectamine 3000 (Thermo Fisher Scientific). Thirty hours post transfection, *Renilla* and firefly luciferase activities were measured by the Dual-Luciferase Reporter 1000 Assay System (Promega).

### Chromatin immunoprecipitation

Chromatin immunoprecipitation (ChIP) was performed using the Simple ChiP® Plus Sonication ChIP Kit using Y79 cells according to the vendor’s protocol (Cell Signaling Technology). After ChIP-seq library constructed, the library was sequenced using an Illumina HiSeq2000 platform. Primer sequence information for ChIP-PCR is also listed in Supplemental Table S1.

## Supplementary information

Supplemental Table S1

Supplemental Legends

checklist

Supplemental Figure S1.

Supplemental Figure S2.

Supplemental Figure S3.

Supplemental Figure S4.

Supplemental Figure S5.

Supplemental Figure S6.

Supplemental Figure S7.

Supplemental Figure S8.

Supplemental Figure S9.

Supplemental Figure S10.

Supplemental Figure S11.

## Data Availability

The raw RNA-seq data from this study have been submitted to the NCBI BioProject database (https://www.ncbi.nlm.nih.gov/bioproject/) under accession number PRJNA752257.
