# Supplementary material for: E2F1/CKS2/PTEN signaling axis regulates malignant phenotypes in pediatric retinoblastoma
Source: Cell Death Dis. 2022 Sep 12;13(9):784. doi: 10.1038/s41419-022-05222-9 (PMC9468144; doi:10.1038/s41419-022-05222-9)
Supplement: Supplementary file 2 — Supplemental Legends [file 41419_2022_5222_MOESM2_ESM.docx]

**Supplemental Figures**

**Supplemental Figure S1. Significant differences in expression profiles between RB tumor and non-tumor samples.**

1. Scatter plot depiction of an unsupervised PCA of gene expression level of our dataset and Rajasekaran’s dataset [22]. (B) Heatmap of all merged samples based on the expression level of the top 5000 most variable genes. Sample type and sample source are indicted by the top color bar above the columns.

**Supplemental Figure S2. Ten cancer-associated genes were upregulated using RNA-seq data of RB and control samples.** RNA-seq data of RB showed that 10 chosen genes enriched in cancer-associated pathway were all upregulated at different degrees using RB samples compared with normal tissues (Retina).

**Supplemental Figure S3. Hallmark gene signatures associated with retinoblastoma progression.**

(A) GSEA of all significantly enriched gene sets. Positive normalized enrichment score means genes from this geneset are enriched in upregulated genes. (B, C, D) Significant enrichment of DNA repair (B) and MYC targets (C) and mitotic spindle (D) in the retinoblastoma compared to normal samples.

**Supplemental Figure S4. Differential expression of four transcription factors potentially bound to the promoter region of *CKS2*.** FPKM, Fragment Per Kilobase of transcript per Million mapped reads based on RNA-seq data. Each data point represents a sample. ***, P < 0.001; **, P < 0.01, by two-tailed *t*-test.

**Supplemental Figure S5. Sanger sequencing diagrams of *CKS2*’s promotor with different TFs’ potential binding sites replaced by a palindromic sequence are shown with blue background.**

**Supplemental Figure S6. Luciferase activity of *CKS2*’s wild-type (WT) and mutated (Mut) promotors in 293T cells.** The mutation was achieved by replacing a palindromic sequence to a specific TF potential binding site.

**Supplemental Figure S7**. **Based on GEPIA, *E2F1* had significantly higher expression in multiple cancer types when comparing tumor to normal tissues.** The red and green lines within a rectangle represent tumor and normal tissues, respectively. Median expression values are labeled as the crossed black short line. Increased and decreased expression in tumor compared with normal tissue are shown as red and green font, respectively. TPM, transcript per million mapped reads based on RNA-seq data.

**Supplemental Figure S8**. **Based on GEPIA, *CKS2* had significantly higher expression in multiple cancer types when comparing tumor to normal tissues.** The red and green lines within a rectangle represent tumor and normal tissues, respectively. Median expression values are labeled as the crossed black short line. Increased and decreased expression in tumor compared with normal tissue are shown as red and green font, respectively. TPM, transcript per million mapped reads based on RNA-seq data.

**Supplemental Figure S9**. **A ChIP-seq peak of E2F1 located within one kb upstream of *CKS2* transcription start site in MCF7 cells.** The blue arrows represent the transcription direction. The ChIP-seq data is from ENCODE dataset (TF ChIP-seq of MCF-7) and ChIP-seq track is visualized by Integrative Genome Viewer (IGV).

**Supplemental Figure S10. Knockdown of *CKS2*** **delayed cancer-associated phenotypes in WERI-Rb-1 cells.** (A) Knockdown of *CKS2* in WERI-Rb-1 cells evaluated by Western blotting. (B) Proliferation rate evaluation of WERI-Rb-1 without (Scramble and Ctrl) and with *CKS2* knockdown (*CKS2*-KD1 and *CKS2*-KD2) by CCK-8 assay. (C) WERI-Rb-1 cells were seeded in six-well-plates at 1,000 cells per well for colony formation assay. (D) EdU staining assay of WERI-Rb-1 cells without (Scramble and Ctrl) and with CKS2 knockdown (*CKS2*-KD1 and *CKS2*-KD2).

**Supplemental Figure S11. Knockdown of *CKS2* increased *PTEN* expression in Y79 cells based on RNA-seq data.** (A) Lentivirus knockdown efficiency of *CKS2* in Y79 cells evaluated by RNA-seq data. (B) *PTEN* expression in *CKS2*-KD Y79 cells validated by RNA-seq data.
