# Supplementary figures and images for: E2F1/CKS2/PTEN signaling axis regulates malignant phenotypes in pediatric retinoblastoma
Source: Cell Death Dis. 2022 Sep 12;13(9):784. doi: 10.1038/s41419-022-05222-9 (PMC9468144; doi:10.1038/s41419-022-05222-9)

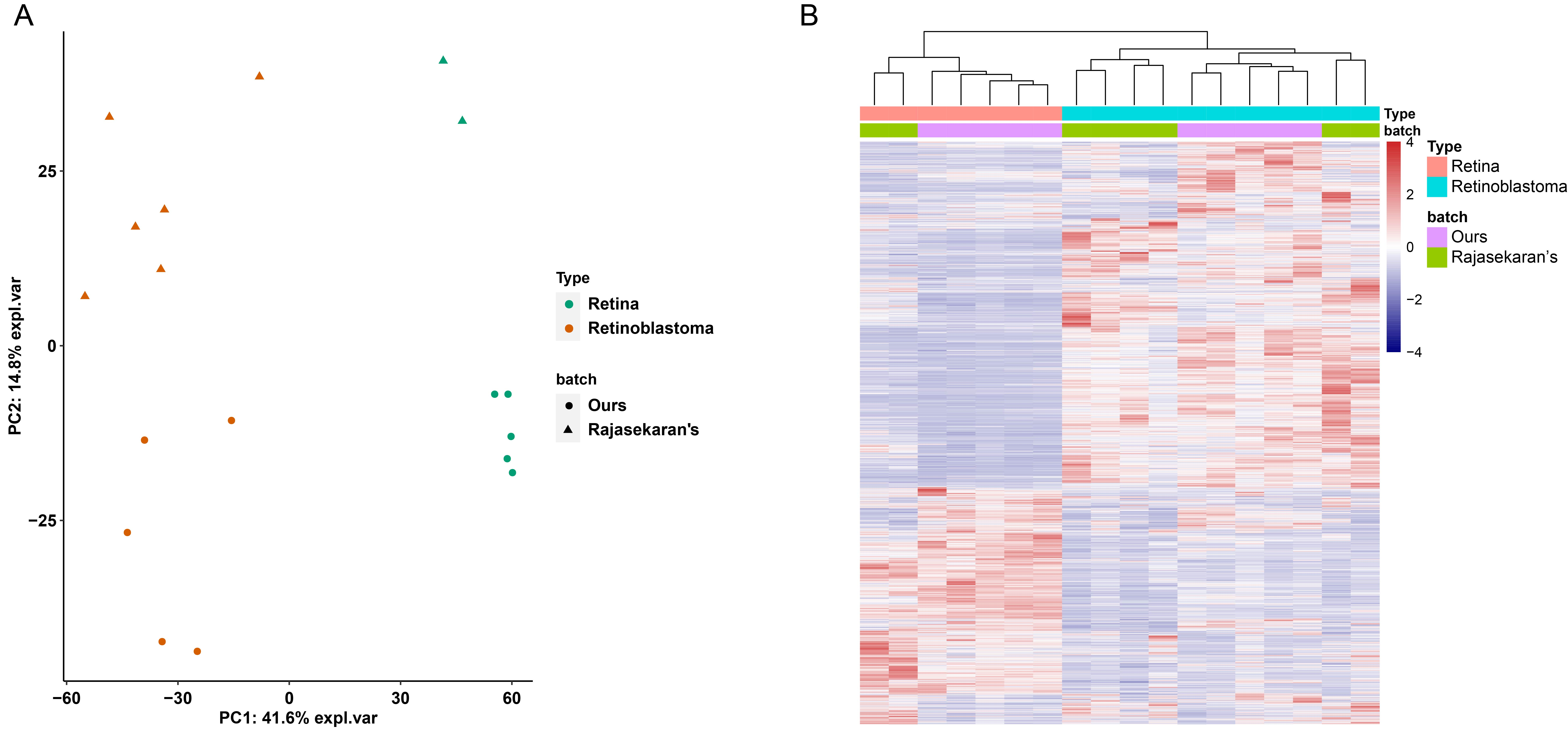

Supplement: Supplementary file 4 — Supplemental Figure S1. [file 41419_2022_5222_MOESM4_ESM.png]

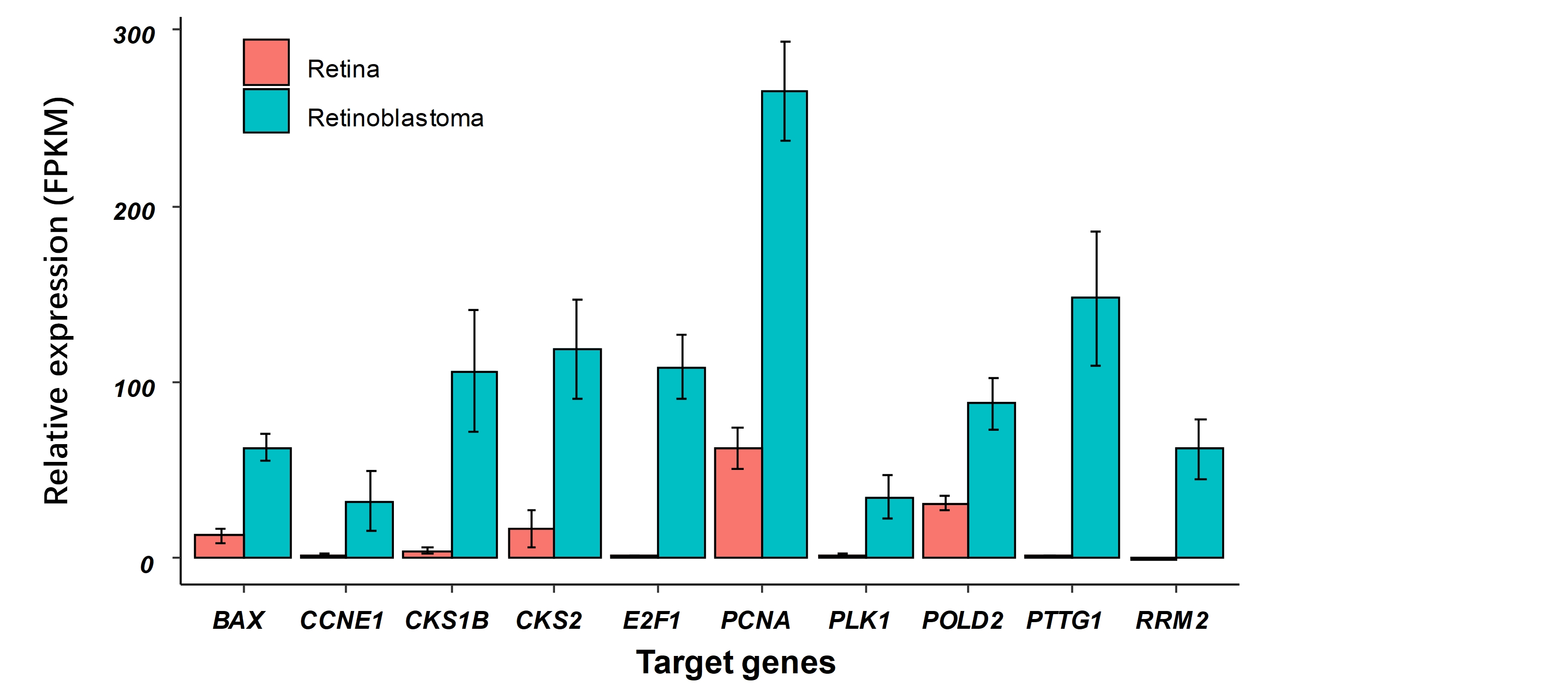

Supplement: Supplementary file 5 — Supplemental Figure S2. [file 41419_2022_5222_MOESM5_ESM.png]

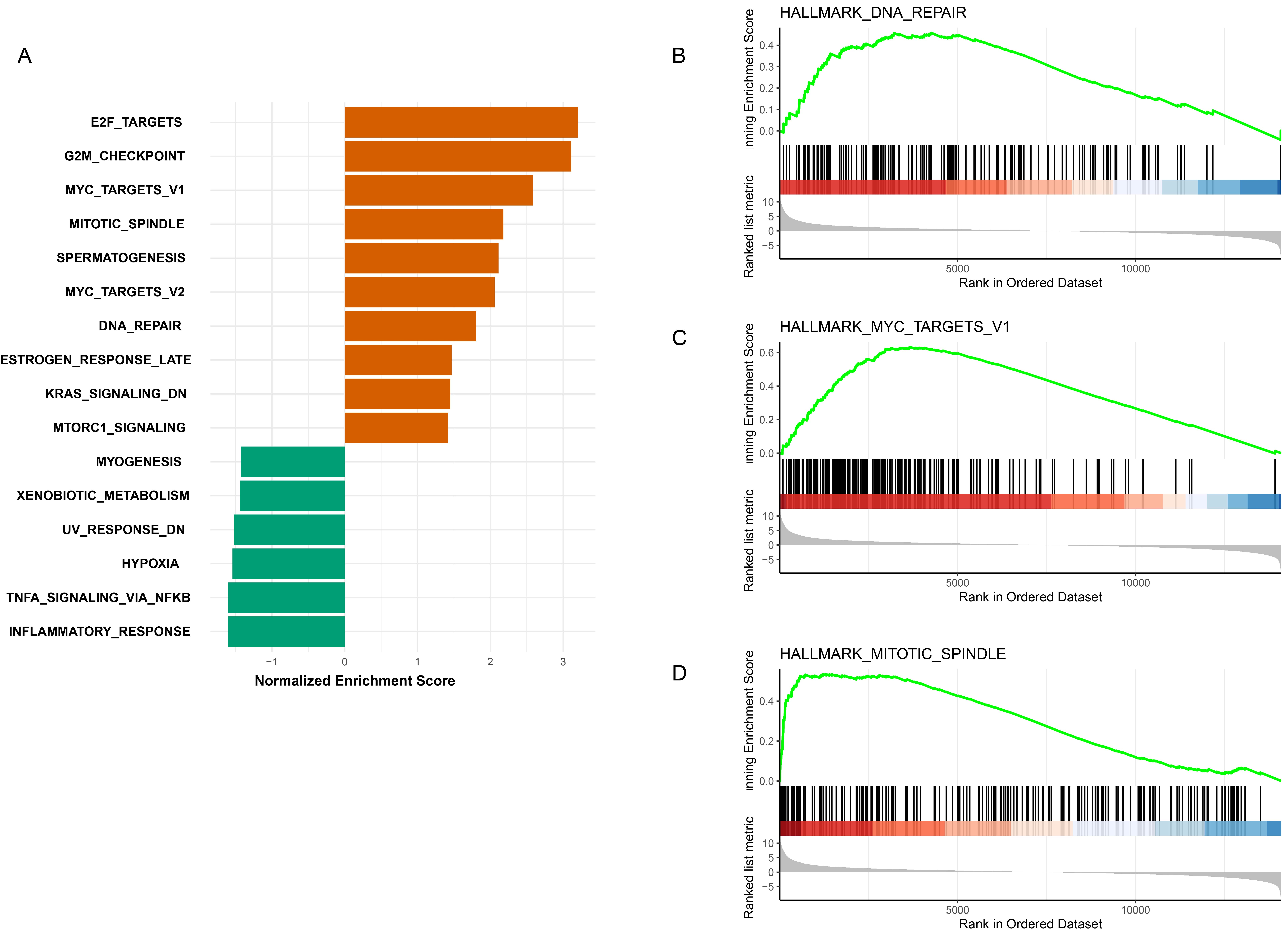

Supplement: Supplementary file 6 — Supplemental Figure S3. [file 41419_2022_5222_MOESM6_ESM.png]

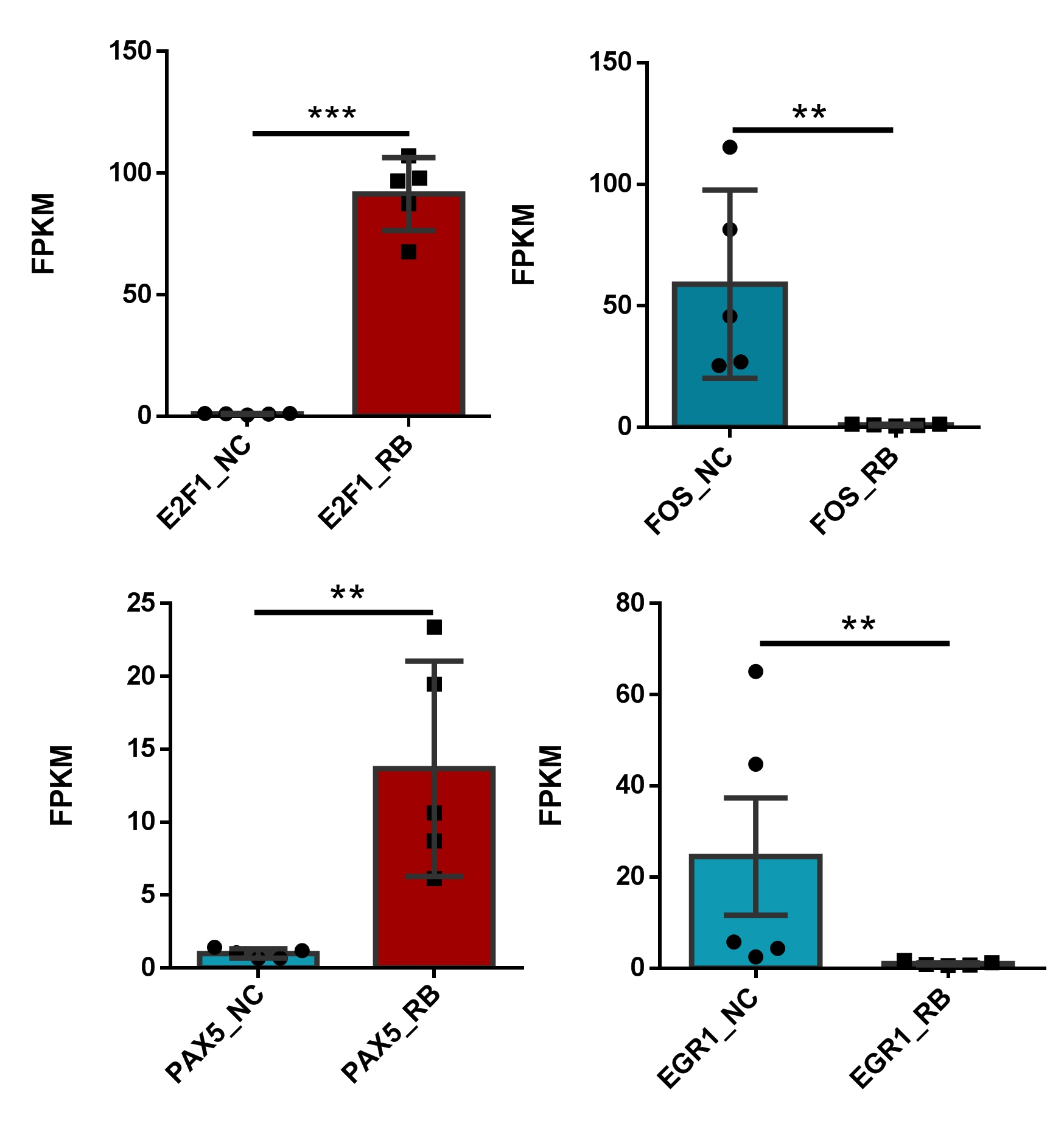

Supplement: Supplementary file 7 — Supplemental Figure S4. [file 41419_2022_5222_MOESM7_ESM.png]

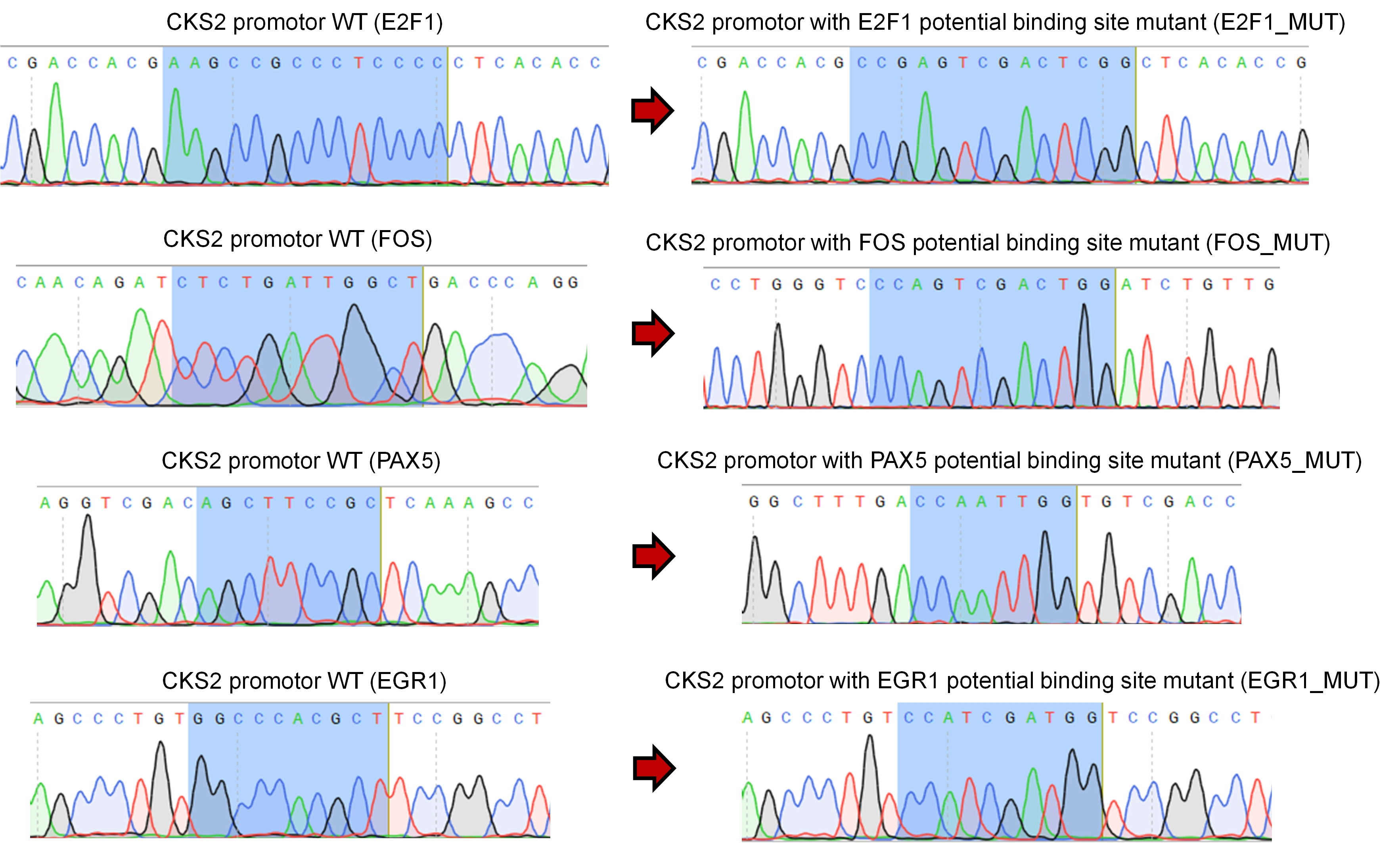

Supplement: Supplementary file 8 — Supplemental Figure S5. [file 41419_2022_5222_MOESM8_ESM.png]

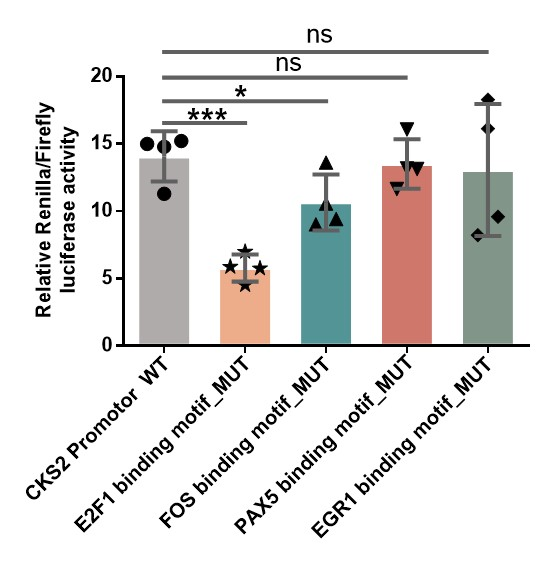

Supplement: Supplementary file 9 — Supplemental Figure S6. [file 41419_2022_5222_MOESM9_ESM.png]

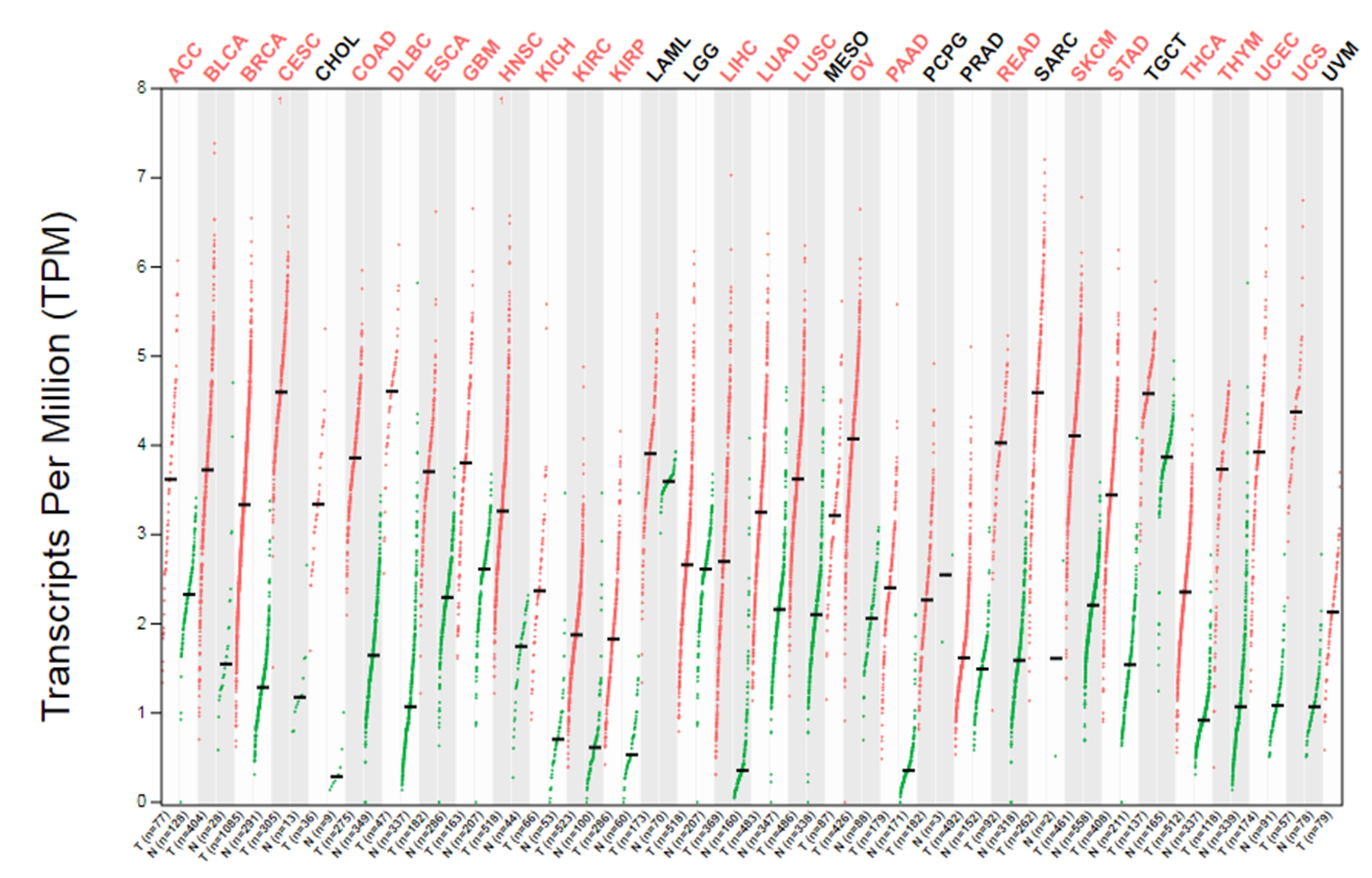

Supplement: Supplementary file 10 — Supplemental Figure S7. [file 41419_2022_5222_MOESM10_ESM.png]

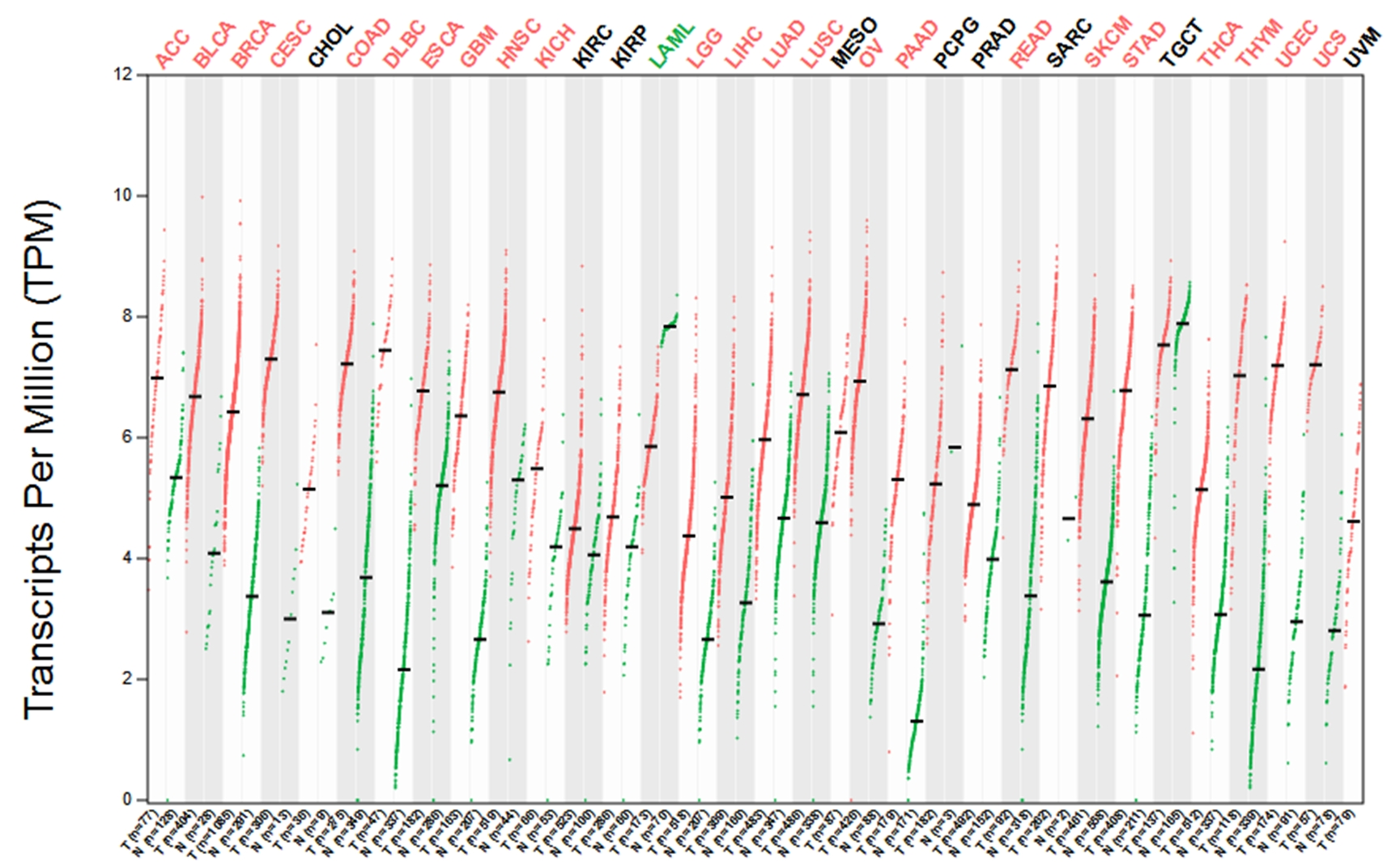

Supplement: Supplementary file 11 — Supplemental Figure S8. [file 41419_2022_5222_MOESM11_ESM.png]

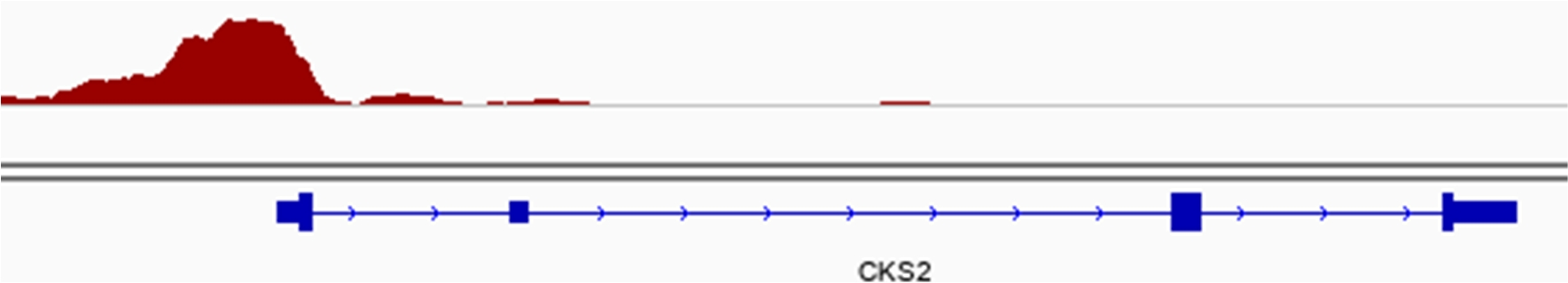

Supplement: Supplementary file 12 — Supplemental Figure S9. [file 41419_2022_5222_MOESM12_ESM.png]

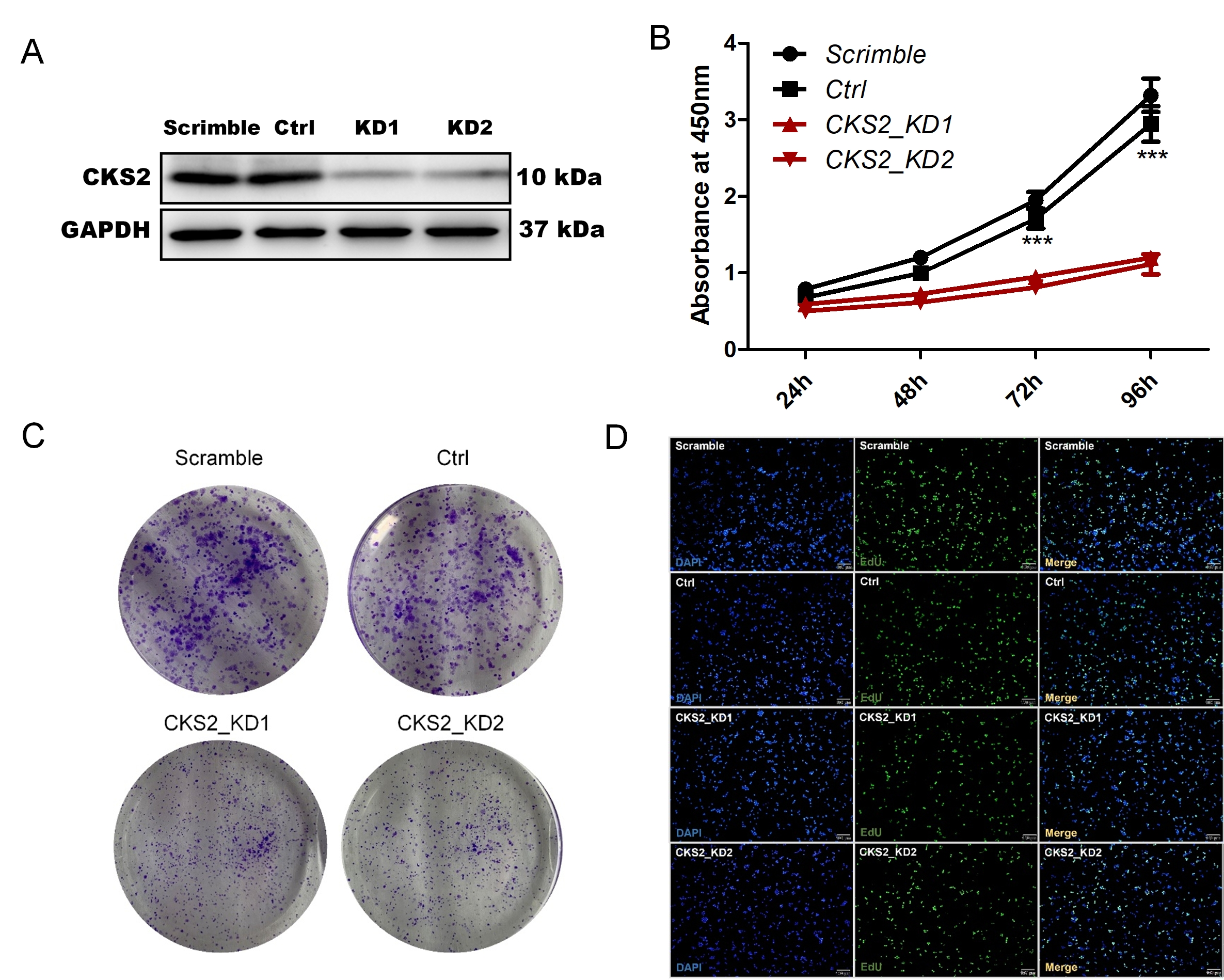

Supplement: Supplementary file 13 — Supplemental Figure S10. [file 41419_2022_5222_MOESM13_ESM.png]

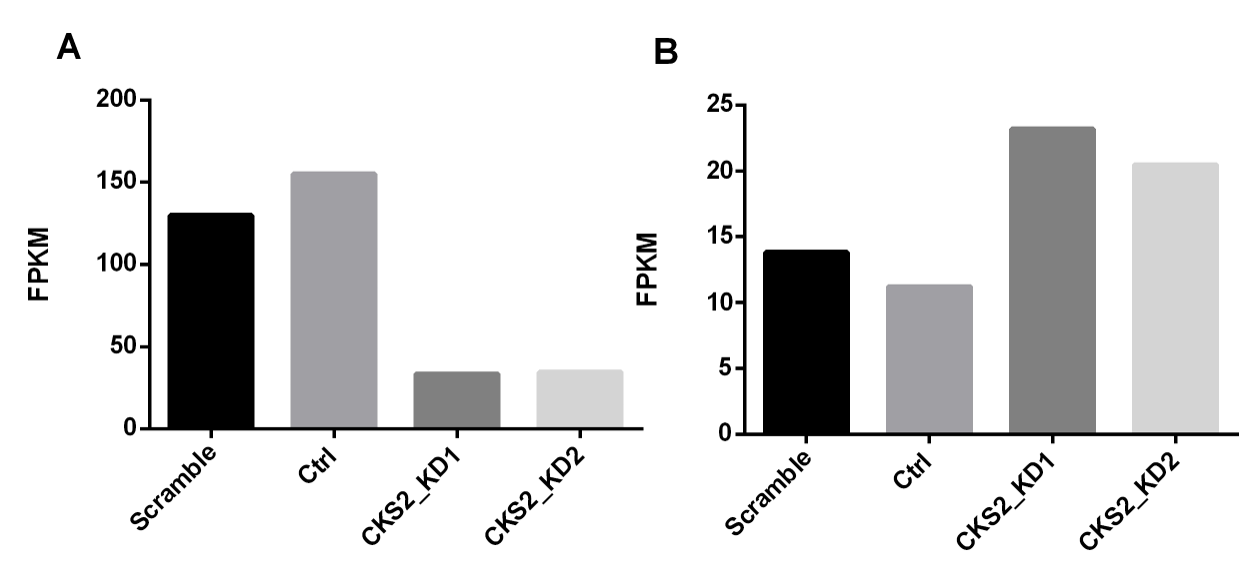

Supplement: Supplementary file 14 — Supplemental Figure S11. [file 41419_2022_5222_MOESM14_ESM.png]
